# Qualitative Olfactory Disorders: Patient Experiences and Self-Management

**DOI:** 10.1177/21526567211004251

**Published:** 2021-09-22

**Authors:** Carl Philpott, Joanne Dixon, Duncan Boak

**Affiliations:** 1 The Norfolk Smell & Taste Clinic, James Paget University Hospital, Gorleston, UK; 2 Norwich Medical School, University of East Anglia, Norwich, UK; 3 Fifth Sense, Barrow-on-Furness, Cumbria, UK

**Keywords:** parosmia, phantosmia, qualitative olfactory disturbances, olfactory dysfunction

## Abstract

**Background:**

Qualitative olfactory disorders in the form of parosmia and phantosmia are very subjective and cannot be measured at present. They pose an unpleasant experience for patients and a therapeutic challenge for clinicians.

**Objective:**

This study aimed to characterise the specific experiences of patients affected by the qualitative symptoms of parosmia and phantosmia including both triggers for symptoms and self-help measures they have tried.

**Methods:**

A cross-sectional survey questionnaire was developed with the input of patient experts within the charity Fifth Sense. The survey was then open online for 3 months to charity members complaining of qualitative symptoms. The survey captured the frequency and impact of symptoms and self-management undertaken. Reflective feedback was also captured from a patient workshop.

**Results:**

There were 100 participants; 61% female, age range 13-88. Common self-reported aetiology included sinonasal disease (17%), idiopathic (33%) and post-viral olfactory loss (26%) and post-traumatic olfactory loss (23%). Parosmia was reported as a daily symptom in 67% compared to 31% for phantosmia; 36% complained of suffering with both symptoms. Only 4% of respondents reported having received any successful treatment for their qualitative symptoms and 58% reported having received no treatment whatsoever. Olfactory training was the most common self-management method reported.

**Conclusion:**

This study illustrates that qualitative disturbances remain problematic for those who experience them due to the duration of symptoms, the relative lack of experience or knowledge amongst medical professionals and the lack of therapeutic options. In future, consideration needs to be given to adaptation and coping strategies to help patients deal with these symptoms.

## Introduction

### Background and Rationale

Whilst we are now witnessing the rise of sudden onset anosmia as a marker of Covid-19 coronavirus infection,^1–7^ it may serve to remind us that at least an estimated 5% of the population^8^ are affected by olfactory dysfunction. These disorders can be both quantitative and qualitative in nature. Common causes of olfactory disorders include chronic rhinosinusitis, post-infectious olfactory dysfunction and post-traumatic olfactory dysfunction^9^ with neurological diseases such as Parkinson’s sometimes culpable, but with about 10% of all cases in the community appearing to be idiopathic in nature. Amongst those cases typically common in specialist clinics are post-infectious and idiopathic cases.^10^ The qualitative aspects of olfactory disorders in the form of parosmia and phantosmia are however less well publicised.^11^

Fifth Sense, the UK charity for people affected by smell and taste disorders, was founded in 2012 and working together with patients our previous work has been able to demonstrate the very significant impact of olfactory disorders on those affected.^12,13^ Whilst recent findings show that qualitative disorders may have a positive prognostic significance for the recovery of olfactory disorders^14^ they do however present a particular source of distress to those affected whilst present. Previous studies of patients presenting with olfactory dysfunction have shown that parosmia was present in 34% and phantosmia present in 12% of patients presenting to a clinical setting.^15^ In some of our earlier work, 2 years after the inception of our charity, our membership reported parosmia in 19% and phantosmia in 24% of cases.^12^

Even more so than quantitative olfactory loss, qualitative disturbances provide a source of frustration for both the patient and the clinician. Evidence for prescribed treatment for qualitative disorders was limited to 7 studies in a previous systematic review for phantosmia^16^ and evidence for self-help measures appears to be very limited.^17^ Some patients are so severely affected that consideration has been given to surgical removal of the olfactory epithelium.^18^

### Objectives

This study aimed to characterise the specific experiences of olfactory disorder patients affected by the qualitative symptoms of parosmia and phantosmia including both triggers for symptoms and self-help measures they have tried.

## Materials and Methods

### Study Design

The study was designed as a cross-sectional survey of the personal experience of people affected by qualitative olfactory disorders in living with and managing these symptoms. A survey questionnaire was developed using a clinician-expert patient partnership. The survey ran online and was available for a period of 6 months. It was promoted via social media internationally using the Fifth Sense links. One of our charity’s volunteers (JD) also provided a personal account of her experience of parosmia as well as inviting comments from her local support group in Newcastle.

### Setting and Participants

The survey was open to anyone globally with access to the world wide web and declaring themselves as someone affected by the symptoms of parosmia and phantosmia, regardless of the aetiology. Survey participants were able to access the survey themselves free of charge via the web-based platform SurveyMonkey. Participants were self-selecting and could participate from any country internationally.

### Data Sources/Management and Variables

The survey asked for basic demographics including age and sex. The terms parosmia and phantosmia were then defined. Participants were asked to declare the underlying cause for their smell loss. Further questions explored the timing, intensity, frequency and impact of qualitative symptoms along with any triggers and self-management undertaken.

### Bias

We aimed to reduce bias in responses by making the survey widely available and it reflects the membership base of charity which is both in the UK population and in other countries. Although patients will be self-selecting in completing this survey, they will represent the patients likely to present to clinicians with qualitative disorders and this survey **is not** designed to estimate prevalence or comment on the relative frequency of qualitative symptoms amongst those with olfactory disorders or the community at large. We do not believe any lack of direct clinician involvement or psychophysical testing detracts from the findings herein.

### Study Size and Statistical Methods

As this was an exploratory study to capture patient experiences, no sample size was set. Due to the nature of the study, descriptive statistics only have been utilised in reporting the survey data.

## Results

### Participants

A total of 100 participants recorded information on the survey during the study period. The number of “non-respondents” is unknown.

### Descriptive Data

Of the 100 participants, 61 were female and 39 were male. The age of participants ranged from 13 to 88, with a mean age of 51. The aetiology reported for participants included 33% reporting idiopathic olfactory loss (IOL), 17% chronic rhinosinusitis (CRS), 23% post-traumatic olfactory dysfunction (PTOD) and 26% post-infectious olfactory dysfunction (PIOD) and 5% iatrogenic; 4 patients reported both PIOD and CRS are included within the above percentages. The range of duration of reported qualitative olfactory disorders was 1 month to 40 years with a mean of 7 years. Eighty percent of respondents reported experiencing parosmia whilst 65% reported phantomsia; 36% of patients reported experiencing both symptoms.

### Main Results

#### Frequency and impact of symptoms

Symptoms of parosmia were reported as occurring more frequently (Table 1), with it being reported as a daily symptom in 67% compared to 31% for phantosmia (Table 2) with an associated higher quality of life impact. The onset of their qualitative symptoms was reported as being sudden in 65%. Parosmia was more likely to be constant (64%) than phantosmia, which was reported as fluctuating in 59%.

**Table 1.** Responses to Question on Parosmia Impact (n/%).

Is your parosmia…	YES	NO	Not Applicable
A daily occurrence?	67	9	24
Intense?	44	27	29
Having an impact on your enjoyment of every day	62	13	25
Is causing you to (or has done) lose weight?	21	51	28

**Table 2.** Responses to Question on Phantosmia Impact (n/%).

Is Your Phantosmia…	YES	NO	Not Applicable
A daily occurrence?	31	31	38
Intense?	33	25	42
Having an impact on your enjoyment of every day	32	29	39
Is causing you to (or has done) lose weight?	10	47	43

#### Self-management of symptoms

Only 4% of respondents reported having received any successful treatment for their qualitative symptoms and 58% reported having received no treatment whatsoever. In those 4 cases where treatment was reported as being successful, 1 had received oral steroids, 1 haloperidol, 1 a polypectomy and 1 acupuncture. There were several comments about topical steroids not helping. Stimulating the nose with other smells (olfactory training) was the most common self-management method (Table 3). Several reported the use of nasal douching, but other options left in the comments included blocking nasal passages with tissue or nasal plugs, using dried eucalyptus leaves in boiling water and inhaling the steam and drinking the tea inhaling the steam, thyme and lavender and castor oil drops in the nose.

**Table 3.** Self-Help Measures Tried by Participants.

Measure Tried	Participants (%)
Valsalva manoeuvre (closing one's mouth, pinching one's nose shut while pressing out as if blowing up a balloon)	33
Head movements	20
Stimulating the nose with other smells (olfactory training)	67
Stimulating the nose with water (nasal douching/rinsing)	39
Stimulating the nose with deep breaths in through the nose	53
Stimulating the nose with menthol/mustard/pepper spray	33

Comments on stimuli included variations between random and constant nature in those with phantosmia, with reports of specific stimuli including increased rhinitis symptoms, cooking smells, emotional or tense situations, humidity and temperature changes, exercise, memories, visual cues, eating and fatigue; key non-olfactory stimulants are listed in Table 4.

**Table 4.** Non-Olfactory Stimulants That Provoke Symptoms.

Stimulant	Participants (%)
Flushing the nose	2
Pungent smells (trigeminal effect)	17
Sneezing	15
Changes in air pressure	25
Air travel	3
Physical exercise	5

### Personal account

Having suffered an extremely bad cold in 2015 (aged 44) I was unaware of the putrid bad smell and taste that was soon to follow and consume my daily life. Over the coming weeks this foul smell became so overpowering that sleep was my only escape. I started to notice certain smells i.e. coffee, petrol, smoke, cooked food, perfume, fabric softener intensified the smell of rotten flesh and sewage twentyfold. Washing clothes, using soap, deodorant and shampoo were all revolting and using toothpaste would make me retch. It took over my life and affected my work and personal life in such a way that I was off work for 3 months and avoided being around my partner, going out or seeing friends and family. This bad smell was constant even when there were no other odours present. The only place I could stop the smell from intensifying was at home by avoiding certain triggers as mentioned above.

I saw my GP over several months who prescribed a variety of medication none of which worked. To my amazement one GP suggested I stand on my head to clear my sinuses. My nostrils became very dry and crusts developed alongside nose bleeds. Eventually I came across a local ENT consultant in the region who took an interest in these conditions and my GP made an appointment for me, but this would not be for another 6 months. Having explored so much medication without success I became so desperate that I arranged to see this consultant privately and was seen within days. Unfortunately, they confirmed that I was suffering from parosmia but could not tell me if I would regain my smell and taste and there was nothing more that could be done. At that point I became hysterical and totally broke down - he recommended I speak to my GP about antidepressants. I left his office distraught and feeling my life was over – I could not bear this constant repulsive smell and taste any longer. Thankfully a couple of days later my consultant called who’d spoke with a colleague and recommended I be prescribed with Theophylline. He explained there was no evidence to prove it would be successful as it was a trial drug which was used for asthma patients. After 1-2 weeks of taking this medication I found it had suppressed the bad smell to a degree where I could manage. I also noticed that mucus returned in my nose as things slowly started to improve but by this time I had a perforated septum by blowing my nose so hard and dislodging the crusting in order to help breathe. Having read about a case in the US regarding Gabapentin I decided to ask my GP if I could also be prescribed this medication and over time I felt that this helped improve my taste.

Parosmia is a dreadful condition which seriously impacted my mental health and quality of life. However, some people do recover – I have! It may have taken 5 years, but I can say I have 85% of my normal smell and taste back. Having spoken to other people with the same condition it is awful to hear that some GPs and Consultants are uninterested and clinical in telling patients “If your smell doesn't return after 6 months then it's not likely to.” In my experience GPs/Consultants should be supportive and monitor and measure their patients’ progress. They should be made aware of specific medication that can potentially aid recovery and save on cost spent on medication that will have no effect. There should also be more research done on trial drugs.

I fully understand that the medical profession is unable to prove that a patient can recover from parosmia but equally there aren't any statistics to say that a patient won't recover either. Therefore, taking an interest and having a positive approach will support patients on their potential journey to recovery or at least give them time to slowly adapt and find coping mechanisms.

### Feedback From Fifth Sense Newcastle Hub Members

- Depression/anxiety when living alone, afraid to go out with friends for fear of eating something that suddenly causes a serious chemical taste/smell and I have to leave. The taste e.g. dressings with vinegar - lemon juice. Loss of joy of smelling cut grass, sea, flowers, personal contact. Life is so bland - feel alien.
- It is depressing that I might have to suffer with this for the rest of my life. My GP tells me that considering the health problems of most of his 75-year-old patients my problem is minor. I know it’s not life threatening but it does seriously affect my quality of life.
- My consultant seemed spectacularly uninterested and spent just a few minutes with a cursory examination to then tell me that there was 'no hope' for me! Crushed, I all but gave up, and was then assaulted by a further issue where my no-taste was largely replaced with an unpleasant sensation/taste for most foods.
- I can't live with this condition - having this foul smell and taste every second of the day is just unbearable.
- The condition manifests itself for me by fairly extreme and unpleasant smells/tastes - somewhat akin to the sharp, sour smell of vomit - for several types of foods (coffee, beef, eggs, broccoli etc) - but all those different foodstuffs have the SAME unpleasant odour!! Weird in the extreme! And….occasionally, things I could tolerate before, I suddenly become unable to? And, yes, like the leaflet indicates, I feel that I was “better off” when I couldn't smell or taste anything!

## Discussion

### Key Results

Qualitative olfactory disorders can have a significant impact on patients and given the mean duration of symptoms in this cohort was 7 years, indicates the effects may be long-lasting in some of those affected. A lack of either treatment or of effective treatment was evident in 96% of respondents. The combination of these two factors reveals a group of patients with a poor quality of life and the potential for adverse mental health. Many triggers exist but whilst external odours do stimulate symptoms, they are also a means for patients to try to moderate their symptoms.

### Limitations

The survey will not have been seen by those who are not online or do not have access to the aforementioned social media. This is likely to have disproportionately affected the older generations. The charity membership and survey respondents will also tend to be more likely to be those who have more persistent symptoms and perhaps are more resistant cases. The diagnoses were self-reported, and no physical examination or psychophysical olfactory testing was conducted due to the nature of the study setting; of course, parosmia and phantosmia cannot be measured. We believe the responses on the survey genuinely reflect the experiences of our charity’s members, based on our previously published work^12,13^ and the feedback during our members conferences and workshops.^19^ Furthermore, the respondents are likely to represent the patients who have persistent and unresolved symptoms and seek help and advice from clinicians, so discussing the absence of direct clinical assessment misses the point of this study which about patients’ experiences in managing these olfactory disorders.

### Interpretation

The demographics and aetiology of study participants was in keeping with the typical female predominance seen in other studies but interestingly with idiopathic and CRS cases accounting for the aetiology of as many of the respondents as PIOD and PTOD cases. Previous studies have reported the prevalence of qualitative disorders as most frequent in patients with PIOD (occurring in 56% of PIOD cases), with 28% in CRS, 14% in PTOD and 10% in idiopathic cases.^15^ Recovery of qualitative olfactory dysfunction in the study by Reden et al was seen in 29% of those with parosmia after an average of 12 months and in 53% of those with phantosmia. A previous systematic review of the management of phantosmia specifically found a very small evidence base for prescribed treatments that included antipsychotics, antiepileptics, anti-migrainous medications and topical cocaine.^16^ Clearly this group of patients have an unmet need in terms of effective treatments, given that 38% of our participants reported unsuccessful treatment and over half reported no treatment being given. Our case reported above is an example of our experience that theophylline may be a useful therapeutic option to consider but the risks and balances of using an off-licence medication need to be discussed with the patient and more evidence is needed on its role in olfactory disorders.^20^ Gabapentin is also a therapeutic agent to consider but similarly needs further corroboration.

The mean duration of symptoms seen in our study was longer than otherwise reported elsewhere^15,21^ but may reflect the source of recruitment and therefore encapsulating patients whose symptoms persist beyond their immediate clinical contact. The main odorant triggers eliciting parosmia in a study by Pierre et al included petrol, tobacco, coffee, perfumes, fruits and chocolate.^21^ Our volunteers have encapsulated a range of experiences and demonstrate that the experience of parosmia is very much an individual one.

### Generalisability

Qualitative disturbances remain problematic for those who experience them due to the duration of symptoms, the relative lack of interest from medical professionals and the lack of therapeutic options for them. This has a significant impact on their quality of life.^21^ In future, consideration needs to be given to psychological and dietary measures to enable patients to adapt and recover from these disabling distortions, in some ways perhaps akin to tinnitus in the case of phantosmia. There is certainly evidence of the need for more resources for these patients both in terms of direct therapeutic options and indirect supportive options.
